# Endoscopic third ventriculostomy before surgery of third ventricle and posterior fossa tumours decreases the risk of secondary hydrocephalus and early postoperative complications

**DOI:** 10.1007/s10143-021-01570-w

**Published:** 2021-07-22

**Authors:** Pawel Tabakow, Artur Weiser, Malgorzata Burzynska, Przemyslaw Blauciak

**Affiliations:** 1 Department of Neurosurgery, Wroclaw Medical University, Borowska str. 213, 50-556 Wroclaw, Poland; 2 Department of Anaesthesiology and Intensive Care, Wroclaw Medical University, Borowska str. 213, 50-556 Wroclaw, Poland

**Keywords:** Endoscopic third ventriculostomy, Tumour removal, Secondary postoperative hydrocephalus

## Abstract

Endoscopic third ventriculostomy (ETV) is an effective treatment for obstructive hydrocephalus (HCP) at the level of third or fourth ventricle. To date, there is no consensus regarding its role as intervention preceding the operation of tumour removal. The aim of this prospective open-label controlled study is to assess if ETV prevents secondary HCP after tumour removal and if ETV influences the early results of tumour surgery. The study was performed on 68 patients operated for tumours of the third ventricle and posterior fossa. In 30 patients, ETV was performed several days before tumour removal, while in 38 patients, the tumour was removed during a one-stage procedure without ETV. Patients who did not receive ETV before the tumour removal procedure had a higher probability of developing postoperative HCP (n = 12, p = 0.03). They also demonstrated a substantially higher rate of early postoperative complications (n = 20, p = 0.002) and a lower Karnofsky score (p = 0.004) than patients in whom ETV was performed before tumour removal. The performance of external ventricular drainage in the non-ETV group did not prevent secondary HCP (p = 0.68). Postoperative cerebellar swelling (p = 0.01), haematoma (p = 0.04), cerebrospinal fluid leak (p = 0.04) and neuroinfection (p = 0.04) were the main risk factors of persistent HCP. Performance of ETV before tumour removal is not only beneficial for control of acute HCP but also prevents the occurrence of secondary postoperative HCP and may also minimize early postoperative complications.

## Introduction

Endoscopic third ventriculostomy (ETV) is an effective method for the treatment of obstructive hydrocephalus (HCP) at the level of the third or fourth ventricle [11, 28]. In most cases, obstructive HCP is caused by tumours. There is an ongoing debate in the neurosurgical literature concerning its role as a routine procedure before tumour removal. The majority of studies on this topic were performed in paediatric patients and gave incoherent results, favouring its performance either before posterior fossa surgery [21] or after it, only in cases of persistent HCP [8, 16]. In adult patients with obstructive HCP caused by posterior fossa tumours, the risk of persistent HCP after tumour removal was shown to be even lower than in the paediatric population [15]. Although the general view was not to justify ETV in every patient with HCP and posterior fossa tumour, treatment of patients with posterior fossa tumours without ETV was accompanied by a higher rate of postoperative complications [15]. In this prospective controlled open-label study, we sought to determine if ETV performed before tumour removal was beneficial for patients with non-communicating HCP caused by a tumour compared to the group of patients in whom ETV was not performed. Our primary outcome measure was the lack of persistent HCP after tumour removal. The second outcome measures included the patient postoperative clinical status and the rate of early postoperative complications after tumour resection.

## Methods

**Fig. 1:**
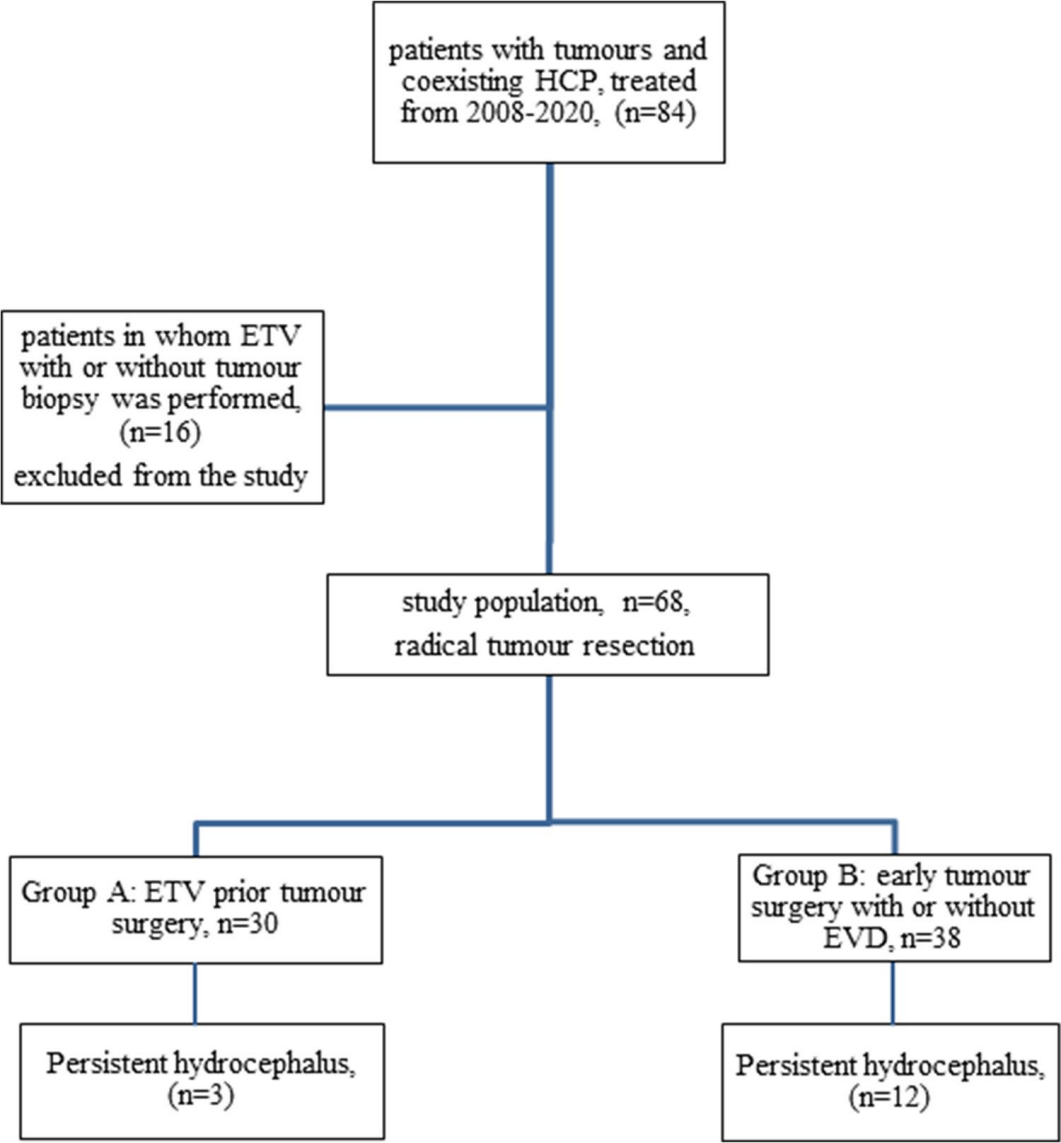
Flow chart describing the study population and the results concerning the primary outcome measure

We prospectively analysed the treatment outcomes of 84 adult patients diagnosed in our department from 2008 to 2020, with a supratentorial or infratentorial tumour causing non-communicating HCP. From all patients meeting the study’s clinical and radiological criteria of active HCP (Evans Index larger than 0.3), we excluded those in whom we only performed ETV with or without a tumour biopsy (n = 16). The remaining patients (n = 68), who qualified for radical tumour removal, were divided into two groups: group A—patients who underwent a two-stage treatment—ETV, followed by tumour removal; group B—only tumour removal with or without external ventricular drainage (EVD) performed during the same surgery (Fig. 1). In all cases, the decision on treatment modality (A or B) was made by the operating neurosurgeon based on his experience. In each case, an informed written consent was obtained from the patient. The study was approved by the Bioethics Committee of the Wroclaw Medical University.

The patients were evaluated clinically using the Glasgow Coma Scale (GCS) and Karnofsky Performance Status (KPS). Patient comorbidities and risk of general anaesthesia were assessed with the ASA Physical Status Classification score. The extent of HCP was evaluated using the Evans Index. In each group, the tumour characteristics included localization (third ventricle/pineal region/posterior fossa; intraventricular (midline) vs extraventricular (peripheral) localization and histological type (benign/malignant). The clinical result after the surgery was assessed using the KPS score, while the extent of HCP resolution was evaluated radiologically. The early postoperative complications were divided into the following groups: neurological deterioration, haematoma, brain oedema, leak of cerebrospinal fluid (CSF), pseudomeningocoele, overdrainage syndrome, ischaemia, infection (wound infection or meningitis) and death. Our primary outcome measure was the lack of persistent HCP after tumour removal, evaluated after the surgery on CT or MRI scans. The second outcome measures concerned patient clinical status and the rate of early postoperative complications.

### Follow-up

The patient postoperative status was evaluated from the day of operation to discharge and at least 6 months after surgery. It included neurological examination and radiological evaluation (head CT and MRI studies). We have focused in the long-term observation not on the oncological aspect of treatment, that in all cases was planned to be as radical as possible, but on the general and neurological status of the patients, as well on the assessment of control of HCP. If direct patient follow-up was not possible, data were obtained via telephone calls with patients or their relatives.

### Statistical analysis

All statistical analyses were performed with TIBCO Statistica™ (version 13.3; TIBCO Software Inc., Palo Alto, CA, USA) either by unpaired and paired two-sample t-tests, the Mann–Whitney U test, the Wilcoxon signed-rank test or chi-square test. A value of p < 0.05 was considered statistically significant.

## Results

A summary of patient data and preoperative clinical status is shown in Table 1. There was no statistical difference between groups A and B in terms of age, gender, Evans Index, GCS, ASA, KPS score and tumour size. The tumour characteristics in terms of localization and histology are summarized in Tables 2 and 3. In both groups, most tumours were found in the posterior fossa and had a predominantly intraventricular extension. The pineal and tectal region, the third ventricle and the thalamus were the next common areas of tumour localization. There were 42 cases of intraventricular (midline) and 26 extraventricular (peripherally localized) tumours. Most of the midline tumours were gliomas (n = 18, ependymoma, astrocytoma etc.), followed by metastatic tumours (n = 14), pineal gland tumours (n = 6) and medulloblastomas (n = 5). Extraventricular tumours were represented by acoustic schwannomas (n = 7), meningiomas (n = 6), metastatic tumours (n = 3), vascular tumours (n = 3) and single cases of high-grade glioma, lymphoma, cholesteatoma and pineal region tumour.

**Table 1:** Patient demographic and clinical characteristics. *ASA* American Society of Anaesthesiologists Physical Status Classification score, *GCS* Glasgow Coma Scale; *KPS* Karnofsky score, *EI* Evans Index; group A—tumour surgery preceded by ETV; group B—early tumour surgery without ETV

	Group A	Group B	p value
Number of patients	30	38	
Age	38.7	47.6	0.10
Female	15	17	0.66
Male	15	21	0.66
ASA	2.17	2.41	0.23
GCS	14.3	14.5	0.17
KPS	70	76.3	0.52
EI	0.35	0.33	0.12
Tumour size	19.54 cm^3^	25.75 cm^3^	0.13

**Table 2:** Tumour characteristics in terms of location. Group A—tumour surgery preceded by ETV; group B—early tumour surgery without ETV

Location	Total	Group A	Group B
Infratentorial	52	19	33
Pineal region	10	8	2
Tectal region	2	2	0
Thalamopeduncular region	4	1	3

**Table 3:** Tumour general histopathological characteristics.*Haemangioblastoma, haemangiopericytoma; group A—tumour surgery preceded by ETV; group B—early tumour surgery without ETV

Histopathology	Total	Group A	Group B
Intraventricular	48	22	26
Extraventricular	20	8	12
Gliomas -Low grade -High grade	19 15 4	8 6 2	11 9 2
Metastases	17	6	11
Primary pineal gland tumours	7	7	0
Schwannoma	7	4	3
Meningioma	6	0	6
Medulloblastoma	5	3	2
Vascular tumours*	4	2	2
Cholesteatoma	1	0	1
Lymphoma	1	0	1
Arachnoid cyst	1	0	1

### Primary outcome measure—development of secondary HCP

**Fig. 2:**
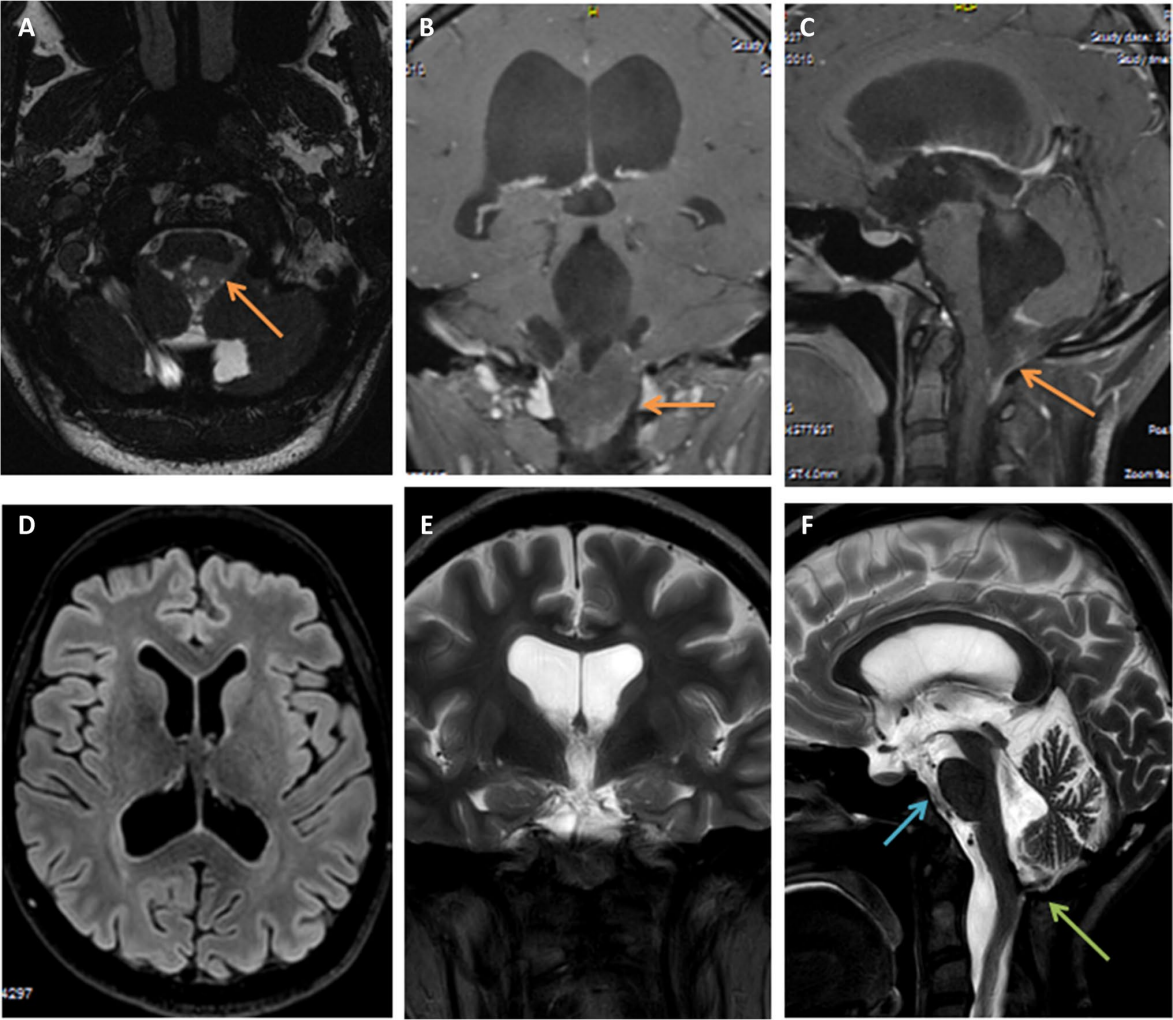
MRI scans performed in a 26-year-old woman with a 4th ventricle subependymoma. (**A**), (**B**) and (**C**) show axial fiesta-3D and contrast-enhanced T1-weighted coronal and sagittal images. Orange arrows show the tumour obliterating the foramina of the 4th ventricle with a subsequent obstructive four-ventricle HCP. (**C**), (**D**) and (**F**) demonstrate the FLAIR axial and T2-weighted coronal and sagittal MRI images after 10 years following ETV and tumour resection. Note that there is no tumour visible and no evidence of HCP (a marked decrease in Evans Index). The blue arrow in picture (**F**) shows the flow void phenomenon across the area of ventriculostomy, while the green arrow points to areas of possible postoperative adhesions (in black) at the level of the foramen of Magendie

In the ETV group (A), only 3 out of 30 patients (10%) developed HCP after the tumour resection surgery. In one patient, HCP was treated 24 days after surgery, while in the remaining 2, after a mean period of 161 days. Most patients continued to be HCP-free after ETV, and subsequent tumour removal during the median follow-up of 6 months. Our longest observation periods lasted 90–96 months (Fig. 2).

**Fig. 3:**
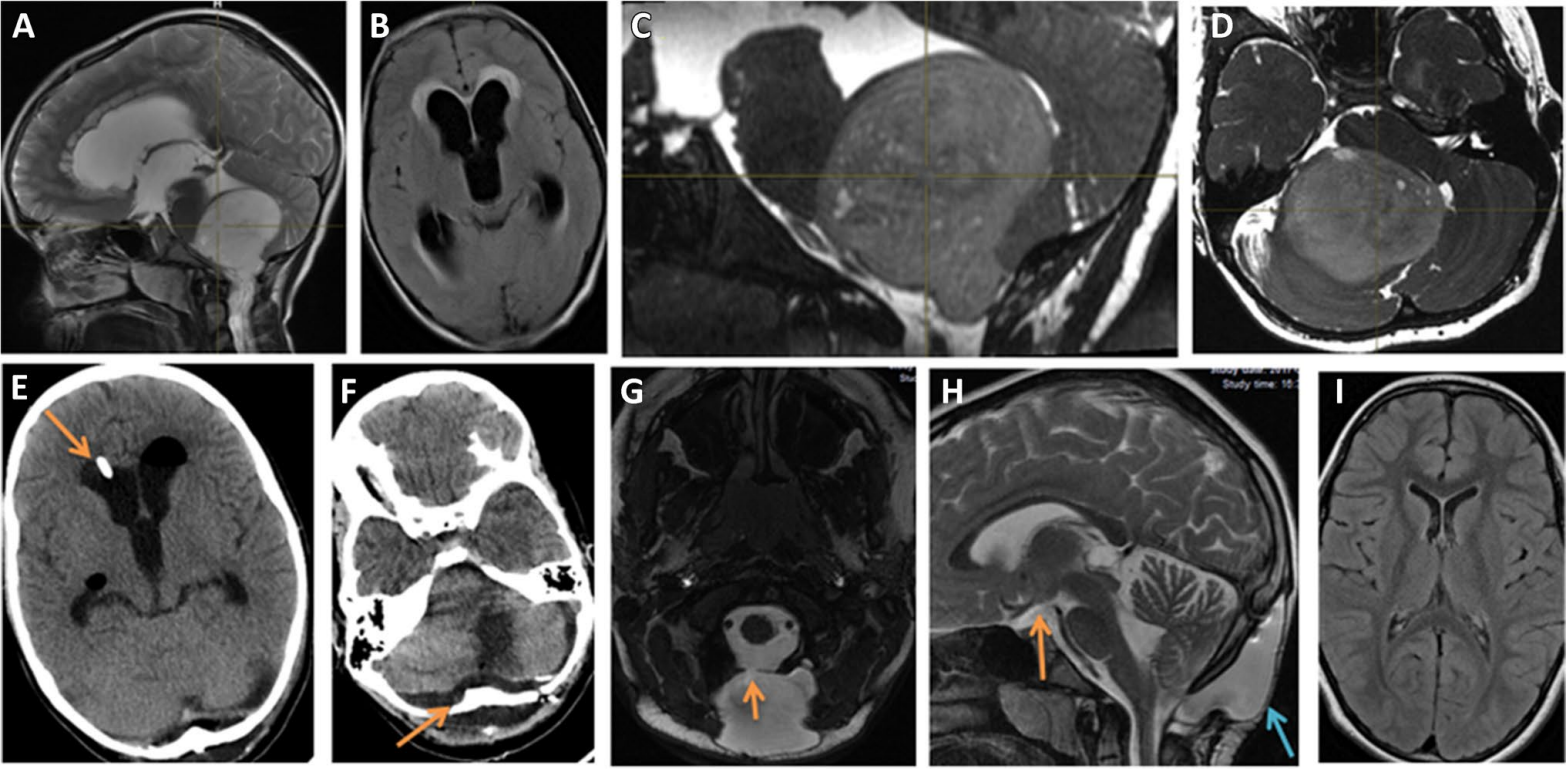
A case of a 7-year-old girl operated for pilocytic astrocytoma of the posterior fossa. (**A**) Sagittal T2-weighted scan shows a large tumour obliterating the 4th ventricle. (**B**) FLAIR axial scan demonstrates active HCP**.** (**C**), (**D**) Fiesta 3D sagittal and axial scans show the tumour compressing the brain stem and cerebellum, restricting the CSF flow. (**E**) Postoperative CT scan performed on day 6 after tumour resection. HCP is still present, although the patient had undergone EVD (orange arrow). For this reason, the patient underwent ETV. (**F**) CT scan from day 6 shows no evidence of tumour in the posterior fossa. However, there is still cerebellar oedema and some CSF collection in the epidural space (orange arrow). (**G**), (**H**), (**I**) MRI scans performed a year after tumour removal. (**G**) A large collection of CSF through a dural fistula is shown on the axial Fiesta 3D image (orange arrow). (**H**) Sagittal T2-weighted image shows the pseudomeningocoele (blue arrow), but, at the same time, good flow of CSF through the area of ventriculostomy (orange arrow) and lack of HCP. A pineal cyst is also present. (**I**) Axial FLAIR scan shows normal ventricle size after ETV performance

**Fig. 4:**
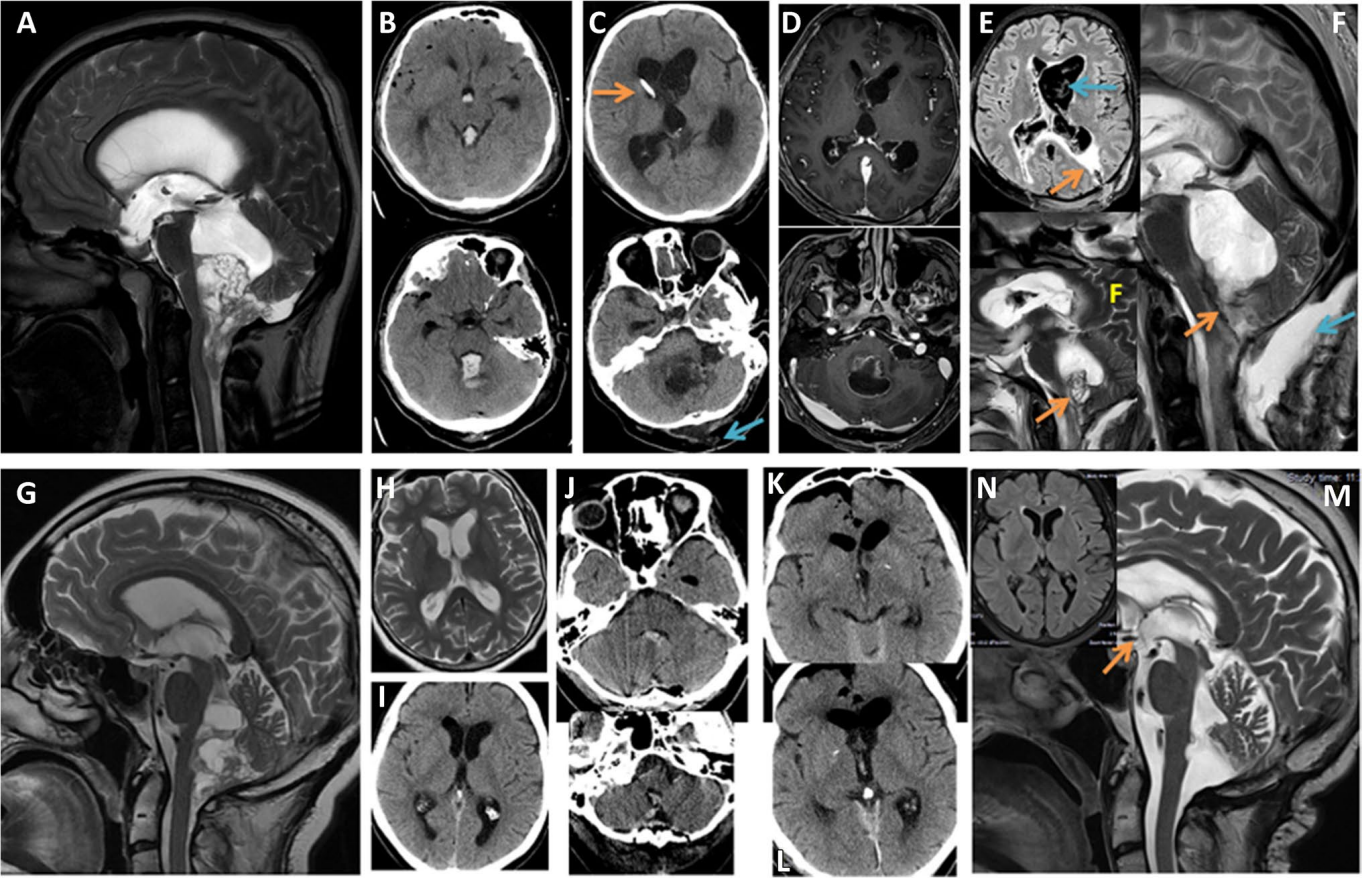
Comparison of outcomes in a 21-year-old man from group B (**A**–**F**) and a 51-year-old woman from group A (**G**–**N**) In both cases, an ependymoma of the 4th ventricle was radically resected, and in both cases, postoperative haematoma was noted. While the course of treatment of patient A was uneventful, the patient from group B continued to deteriorate and died a month after the tumour surgery. (**A**) Sagittal T2-weighted MRI scans show a large tumour obliterating the posterior part of the 4th ventricle and the craniovertebral junction. (**B**) CT images performed on day 1 after tumour resection demonstrate a haematoma obliterating the 4th ventricle, the Sylvian aqueduct and partially the 3rd ventricle. For this reason, the EVD was kept longer, but as the patient did not improve and additionally presented CSF leak, on a postoperative day 7, the haematoma was removed, and duraplasty was performed. (**C**–**F**) CT and MRI scans from day 28 to 29, respectively. In the meantime, the patient underwent 2 additional procedures of EVD replacement and was treated for bacterial meningitis and ventriculitis. (**C**) While no haematoma is visible intraventricularly, there is a marked cerebellar oedema/ischaemia. CSF fistula is still visible (blue arrow). The ventricles are still dilated despite the presence of a ventricular drain (orange arrow). (**D**) Axial T1-weighted contrast-enhanced images show HCP and ventricle enhancement due to ventriculitis. (**E**) FLAIR MRI axial scan demonstrates active HCP (orange arrow) and multiple posthaemorrhagic and postinfectious intraventricular fibrous adhesions (blue arrow). (**F**) Sagittal T2-weighted MRI scans show further adhesions at the level of the 4th ventricle (orange arrows) and subcutaneous CSF collection, secondary to the active HCP (blue arrow). After removal of EVD, the patient finally underwent endoscopic removal of the intraventricular blood remnants. Some adhesions were removed, including the ones in the aqueduct, and ETV was performed. Unfortunately, the patient died several days later due to neuroinfection. (**G**) T2-weighted sagittal MRI scan of a patient from group A shows an almost identical 4th ventricle ependymoma. (**H**) Axial T2-weighted scan demonstrates HCP**.** (**I**) CT scan performed after ETV shows some narrowing of the ventricles. The patient underwent tumour resection 9 days after ETV. (**J**) CT images on day 1 after complete tumour resection show some intraventricular blood. (**K**–**L**) Axial CT scans (day 1) show no HCP but only some air accumulation in the frontal horns and subdurally. (M) Sagittal T2-weighted MRI scan performed 10 years after surgery shows no evidence of tumour regrowth, no HCP and evident flow void phenomenon through the area of ventriculostomy (orange arrow). (N) FLAIR axial MRI scan confirms lack of HCP

In contrast, patients from the non-ETV group (B) had a higher probability of developing postoperative HCP than group A (n = 12, p = 0.03). Twelve out of thirty-eight patients (31.5%) developed HCP (Figs. 3 and 4). The performance of external ventricular drainage in 11 patients during the operation of tumour resection did not prevent secondary (postoperative) HCP (n = 4, p = 0.68). In 8 patients, the observed postoperative HCP was diagnosed in the first month after tumour resection. The mean period from the tumour surgery to HCP treatment in this group was 6.5 days. In the remaining 4 cases, the postoperative HCP was diagnosed and treated after a mean period of 112 days. The tumour size did not influence the development of postoperative HCP in either group. In both groups, secondary HCP was treated preferably by EVD or ETV, if HCP was diagnosed during the first days after tumour resection. In contrast, chronic HCP was treated preferably by shunt implantation and more rarely by ETV.

### Secondary outcome measures—patient clinical status and early postoperative complications

There was a notable improvement in the clinical status of group A patients that started shortly after the performance of ETV. The mean increase in the KPS score was 14.82 points (p = 0.00000). According to the KPS, after tumour removal, group A patients demonstrated further improvement of the clinical status from 84 to 86 points, which was not statistically significant. In addition, there were very few early postoperative complications in this group, including neurological deterioration (n = 3) and wound infection (n = 1), neuroinfection (n = 1), haematoma (1) and CSF leak (1) (Table 4). The overwhelming majority of patients from the ETV group were discharged from the hospital in a good general and neurological condition.

**Table 4:** Summary of postoperative complications. While in group A, the number of complications was almost equal with the number of examined patients, in group B, there were a lot of cases where several complications occurred in one patient

Complications	Group A	Group B
Neurological deterioration	3	14
Infection	2	4
CSF leak	1	7
Haematoma	1	6
Severe brain oedema	-	5
Cerebral ischemia	-	2
Pseudomeningocele	-	2
Overdrainage syndrome	-	1
Death	-	4
Total number of complications	7	45
Number of patients with complications	5	20

In contrast, non-ETV group patients (B) demonstrated a significant decrease in the postoperative KPS score (p = 0.004) (KPS = 69;) compared to ETV group patients (KPS = 86). However, before tumour removal, the KPS score was similar in both populations (group A = 70, group B = 76, p = 0.12). Additionally, there was a markedly higher rate of early postoperative complications in group B (45 complications in 20 patients) (Table 4). The percentage of cases with complications in group A was 16.6% (5/30), while in group B, it was 52.6% (20/38) (p = 0.0023). Complications like marked cerebral/cerebellar oedema or ischaemia, pseudomeningocoele, overdrainage syndrome and death related directly to the neurosurgical intervention were observed only in this group (Table 4, Figs. 3 and 4). In both groups, the tumour size did not influence the clinical outcome and the rate of complications after the resection.

## Discussion

The main goal of this prospective open-label controlled study, performed on patients diagnosed with tumours causing non-communicating HCP, was to evaluate if ETV conducted before tumour removal was beneficial for the patients compared to the group in whom ETV was not performed. Firstly, we sought to determine if ETV could lead to permanent resolution of obstructive HCP, which can develop postoperatively even in the absence of tumour [7, 16]. In addition, we attempted to assess if ETV may have other beneficial influence on the procedure of tumour resection. To evaluate it, we focused on patient clinical status and the rate of postoperative complications, comparing the ETV group (A) with the non-ETV group (B). Both groups were homogenous in terms of the patient numbers, demographic data, preoperative Evans Index, GCS, ASA, KPS score and tumour size (Table 1). Some tumour characteristics like localization were similar, for example, the proportion of tumours with midline-intraventricular versus peripheral-extraventricular localization (Tables 2 and 3). We find this similarity between the groups highly important for the power of our study and the final conclusions. There were also some topographical differences, like more posterior fossa tumours in group B than A (33 vs 19) and more pineal region tumours in group A than B (8 vs 2) (Table 2). The comparison of study groups in terms of tumour histology showed similar results except for the higher number of metastatic tumours in group B (11 vs 6), meningiomas and some rare tumours (cholesteatoma, lymphoma) found only in group B and primary pineal gland tumours present only in group A.

### Development of secondary HCP after resection of tumour causing obstructive HCP

It is generally assumed that if a patient is diagnosed with obstructive HCP caused by a tumour, the radical tumour removal will lead to HCP resolution [8]. Interventions like ETV or EVD and much rarely – implantation of a shunt before tumour removal – are advocated for cases when the patient neurological status is deteriorating, and the tumour cannot be resected in a reasonably short time [1, 17, 18, 20, 24]. These CSF diverting procedures may serve as a ‘safety valve’ to prevent the intraoperative or early postoperative episodes of sudden elevated intracranial pressure [18, 20, 21, 26]. The probability of developing persistent postoperative HCP after tumour surgery in children, in whom ETV was not performed, was reported to be much higher (26.8%) [21] than in adult patients (5.7%) [15]. Our study concerned predominantly adult patients and showed in the group of non-ETV patients a 31.5% rate of developing persistent HCP after tumour surgery, which is comparable to the mean rate of postresection HCP in children with posterior fossa tumours [6, 16, 21]. Postoperative HCP occurred in the non-ETV group (B) mainly within the first month (n = 8). This observation is similar to the findings of other studies [3, 12]. In the ETV group, there was only one patient who developed HCP in the first month. We defined this HCP as early postoperative. The most frequent scenario of early HCP in group B was the presence of cerebellar swelling (5 cases), followed by haematoma in the tumour resection bed (4), CSF leak (3) and single cases of cerebellar and brain stem infarction or bacterial meningitis (Fig. 4, Table 5). The only case of early HCP in group A concerned a patient after pinealocytoma resection, in whom an intraventricular blood clot blocked the area of the third ventriculostomy. Late HCP was diagnosed in 4 group B patients and 2 group A patients. Postoperative adhesions in the area of Sylvian aqueduct or foramen of Magendie (n = 2) or ventriculitis (n = 2) were the causes of late HCP in group B. The factors that led to late HCP in group A were more challenging to predict and included one case of aggressive regrowth of anaplastic astrocytoma of the third ventricle and one case of pachymeningitis in a medulloblastoma patient (Table 5). Our study showed clearly that HCP as a result of cerebellar swelling, haematoma, ischemia or postoperative adhesions concerning the midline paraventricular areas could be avoided if ETV was performed before tumour removal. Group A patients, showing postoperative radiological features of elevated intracranial pressure in the posterior fossa, developed neither early nor late HCP (Fig. 4). The study of Ruggiero et al. strongly supports our findings of the protective role of ETV against acute postoperative HCP due to cerebellar swelling and late postoperative HCP [20]. The performance of EVD in group B patients (n = 11) did not prevent HCP occurrence (n = 4) in all cases. Hence, EVD additionally increased the risk of late HCP in the course of meningitis and ventriculitis due to ventricular catheter contamination or continued CSF leak from the wound, which was observed in 3 patients.

**Table 5:** Summary of the possible mechanisms of development of early and late postoperative HCP in the patients from groups A and B. Note that while in group A, the number of complications leading to HCP was equal with the number of patients with HCP, the number of complications leading to HCP in group B exceeded the number of patients with HCP

	Early postop. HCP	Late postop. HCP
Group A: ETV	-Obstruction of area of ventriculostomy (n = 1)	-Malignant tumour recurrence (n = 1) -Pachymeningitis (n = 1)
Group B: non-ETV	-Cerebellar oedema (n = 5) -Haematoma (n = 4) -CSF leak (n = 3) -Cerebellar ischaemia (n = 1) -Neuroinfection (n = 1)	-Fibrous adhesions (n = 2) -Neuroinfection (n = 2)

Our findings showed that the performance of ETV in patients with brain tumours and coexisting obstructive HCP before tumour surgery is justified. ETV is an effective and relatively easy procedure associated with minimal perioperative risk [2, 5, 14, 25]. However, the learning curve in this technique is much steeper than in the case of EVD [23, 27]. Having long-term experience in neuroendoscopic procedures, we did not observe any neurological deterioration or other permanent complications after ETV. All group A patients improved neurologically in the period before the radical tumour surgery. The decrease of elevated ICP due to ETV resulted not only in clinical improvement of the patients but also enhanced operating conditions for tumour resection. Surgeons who performed tumour resection in group A reported ‘better surgical conditions’ in terms of good brain relaxation. This phenomenon was also noted in other studies on the role of preresection ETV [4]. In our case, it was helpful, especially during operations of tumours obliterating the fourth ventricle or the craniospinal junction, where the opening of cisterna magna for cerebellar relaxation was not possible. The observation of improved surgical conditions in the ETV group should be treated with caution, as we could not quantify the degree of the brain and especially cerebellar relaxation. The scales used for intraoperative assessment of brain relaxation rely on the subjective–virtual and tactile estimation of the brain in the supratentorial space [13]. It is not clear to what extent those scales can be applied to the infratentorial space. Placement of EVD was the alternative solution for a decrease of ICP, but this manoeuvre resulted in a less-controlled and sudden reduction of ICP. Although EVD could ensure with time also cerebellar relaxation, the early postoperative clinical results in terms of complications rate (also due to prolonged application of EVD!) and persistent HCP showed EVD to be a less reliable treatment option than ETV. Gopalakrishnan et al. also demonstrated that children who underwent EVD during posterior fossa tumour resection were 2.5 times more likely to develop persistent HCP compared to those treated without EVD (p < 0.001) [9].

It is disputable whether ETV should be performed in every patient with obstructive three- or four-ventricle HCP due to a tumour—both in the patients with severe HCP (EI > 0.4, GCS < 13) and moderate/mild HCP symptoms (0.3 < EI < 0.4, GCS > 13). Ruggiero et al. used ETV only in children showing severe HCP [20]. Other authors performed ETV in all children with a posterior fossa tumour and clinically and radiologically evident HCP, recommending this procedure almost as a routine [21]. Yet, other authors strongly discourage the routine performance of ETV before tumour removal, indicating a rate of persistent postoperative HCP of 11.5% [8], 12% [16], 22% [26] and citing reports of relatively high rates of complications following ETV, ranging between 5 and 20%. They recommend early posterior fossa surgery (within 24–48 h from admission) to prevent HCP and a routine postoperative ETV for cases of persistent HCP [8, 16]. However, both studies were retrospective and did not discuss in detail the preoperative and postoperative neurological status of patients and the specific postoperative complications. We have shown that ETV may significantly improve the final patient clinical status and, in part, decrease the rate of complications after tumour surgery. This issue is discussed in the next paragraph in more detail.

Studies focusing on HCP frequency in adult patients before posterior fossa surgery and the risk of permanent postoperative or newly developed HCP concluded that ETV is not justified in every patient before posterior fossa surgery [15]. Our study, concerning mainly adult patients (85% of the study group), strongly contrasts with the previous one. We showed clearly the beneficial effect of ETV for control of early and late postoperative HCP (group A patients) and, at the same time, the high risk (31.5%, p = 0.03) of developing persistent HCP in the absence of tumour (group B patients). None of our patients presented clinical or radiological symptoms of severe HCP (Table 1). Nevertheless, we do not support the approach of conducting ‘prophylactic’ ETV before tumour surgery. An indication of possible risk factors of developing persistent postoperative HCP is highly desirable. This issue has been widely studied in the children population. It was shown that younger age [3, 6, 12, 16], severity of HCP [6, 12], midline localization [3, 12], tumour type [6, 12, 16, 22], incomplete tumour resection [3], CSF-related infection [3], pseudomeningocoele [3] and usage of dural graft substitutes [3] increase the risk of developing postoperative HCP. Hence, a scoring system predicting the risk of developing HCP in paediatric patients after posterior fossa tumour resection was proposed [19]. In an attempt to determine the risk factors of developing persistent HCP in the group of adult patients with preoperative HCP, we analysed the natural history of postoperative HCP in the non-ETV group (n = 38). We found a strong dependence between postoperative cerebellar swelling (p = 0.001), haematoma (p = 0.04), neuroinfection (p = 0.04) and CSF leak from the posterior fossa (p = 0.04) and the development of persistent HCP. In this study, it was impossible to determine any preoperative risk factors for developing postoperative HCP in the non-ETV group due to the relatively low number of examined patients. Yet, ETV significantly reduced HCP occurrence in the group of patients with intraventricular and posterior fossa tumours (p = 0.03), which means that these factors might be significant predictors of postoperative HCP in larger groups of patients as well.

### Patient clinical status and early postoperative complications

The comparison of patient postoperative clinical status showed a significant improvement in the KPS score in the ETV group (from 70 to 86 pt.) and a KPS score decrease in the non-ETV group (from 76 to 69 pt.). Clinical improvement in group A started shortly after the ventriculostomy (mean increase of 14 pt.) and became insignificant after tumour surgery. This fact shows that ETV is a powerful method of HCP treatment. The significantly lower KPS score in group B was determined by the presence of persistent HCP observed in 31.5% of patients and significantly more postoperative complications in group B than A (52.6% of patients vs 16.6%, respectively).

Several studies focused on the feasibility of ETV in HCP treatment and prevention underline the fact that ETV patients presented fewer postoperative complications after tumour surgery than non-ETV ones [15, 20, 21]. In a large retrospective study on 243 adult patients, Marx et al. showed that the rate of perioperative complications in ETV patients was 22.9 vs 30.8% in the non-ETV group [15]. While the study of Sainte-Rose et al. conducted in 206 children with posterior fossa tumours presented a rate of complications of 25 vs 38%, respectively [21]. Although the increased rate of complications in the non-ETV group did not reach statistical significance in both studies, a set of postoperative adverse events could be outlined that occurred typically in this group of patients. They include CSF leaks and pseudomeningocoele conditions that often lead to wound infections or meningitis [15, 21]. In our study, CSF leak or pseudomeningocoele were typical complications for the non-ETV group and occurred in 9 patients and only in one patient from the ETV group. In most cases, they were the first symptom of persistent HCP and, in 5 cases, led to meningitis and wound infections (group A—1 case; group B—4 cases). Other observed complications such as excessive cerebellar swelling, haematoma or ischemia and neurological deterioration due to cranial nerve injury or injury of the floor of the fourth ventricle or the quadrigeminal plate were observed predominantly in group B. They were chiefly related to the specificity of the tumour and its relation to eloquent brain structures and the applied resection technique. Yet, we cannot entirely exclude the influence of ETV on the smaller number of neurological impairment cases in group A in the mechanism of gradual and physiological decrease of ICP during a mean period of 5 days before tumour resection, leading to increased brain relaxation and decreased mechanical traction or compression of the neural tissue during surgery. This hypothesis requires conducting more detailed studies using objective assessment tools, for example ICP measurements and ultrasonographic CSF pulsation and blood flow measurements or perhaps finite element modelling of the brain viscoelastic behaviour.

There are few limitations to our study. We could not use the available systems for objective patient allocation using the minimization approach [10], because the decision for the type of performed surgery depended on the personal surgical preference. Some surgeons chose the ‘conventional’ approach, as they were not convinced that their patients would benefit from an ETV preceding tumour removal. A similar unbalanced group classification was also described in other large prospective multicentre studies on the same topic [21].

Although patient distribution between group A and B enabled the comparison of two relatively homogenous groups in terms of demographic features, as well as tumour clinical, radiological, topographical and histological characteristics, 15% of patients were children aged between 5 and 15 years. Conclusively, our findings concerned mainly but not solely the population of adult patients. Yet, only 2 out of 12 patients from the non-ETV group (B), who developed persistent HCP, were children. This fact did not influence the conclusions concerning the risk of postoperative HCP for adults. Another limitation of the study was the relatively low number of examined patients, which made it impossible to determine the preoperative risk factors of developing persistent HCP after tumour resection.

## Conclusions

Analysis of the results of this prospective controlled study showed that the performance of ETV in patients with three- or four-ventricle obstructive HCP before tumour removal is a safe and powerful tool for the prevention of persistent postoperative HCP. ETV also contributed to significantly higher KPS postoperative scores and a lower rate of complications compared to the non-ETV patient group. Treatment of patients with a tumour causing obstructive HCP as a one-stage operative procedure (with or without EVD) is more demanding. It bears the risk of worse outcomes regarding a higher rate of complications and developing postoperative HCP. We showed that the performance of ETV before tumour removal is justified, but we could not determine the preoperative characteristics of patients with a high risk of postoperative HCP. A prospective controlled multicentre study performed on a large number of adult patients could enable the determination of strict indications for the performance of ETV before tumour surgery.

## Data Availability

The data generated during this study are available in the article.
